# The use of intervention mapping to guide the development of a school-based intervention to improve emergency medical services activation for stroke

**DOI:** 10.1186/s12889-024-20809-x

**Published:** 2024-11-28

**Authors:** Ekaterina Volevach, Francine Schneider, Hana Maršálková, Robert Mikulik

**Affiliations:** 1 International Clinical Research Center, St. Anne’s University Hospital Brno, Brno, Czech Republic; 2 First Department of Neurology, St. Anne’s University Hospital Brno and Faculty of Medicine, Masaryk University, Brno, Czech Republic; 3 Department of Health Promotion, Care and Public Health Research Institute (CAPHRI), Maastricht University, Maastricht, the Netherlands

**Keywords:** Intervention mapping, School-based, Stroke, Entertainment-education, Emergency medical services activation, Educational intervention

## Abstract

**Background:**

If carried out correctly and without delay, activation of emergency services by stroke bystanders could improve mortality and disability from stroke. This paper describes the development of a school-based intervention using the Intervention Mapping approach. It aims to improve the appropriate activation of emergency medical services for suspected stroke by 12-15-year-old children.

**Methods:**

The development of the intervention was guided by Intervention Mapping approach. The logic model of the problem was created through analysis of the existing literature and semi-structured interviews with stakeholders. Based on these findings, performance objectives and their determinants were determined and matched to create a model for changing emergency medical services activation behavior. Behavior change methods and their practical applications were then determined. Based on them, intervention messages and materials were designed, the intervention was drafted, pretested, and finalized.

**Results:**

It was found that the main performance objectives for the activation of emergency medical services were (1) recognizing symptoms, (2) communicating with the victim, and (3) calling an ambulance immediately. Their main determinants were knowledge, social influence, risk perception, self-efficacy, outcome expectations, and skills. Determinants were then matched with performance objectives to create the matrices of requested behavior changes. The following change methods were chosen: modeling, elaboration, belief selection, providing cues, scenario-based risk information, and cultural similarity. Methods were translated into practical applications in the form of a short educational film. The production company created, pretested, and finalized the film. As a result, a 5-minute entertainment-education video was created modeling an acute stroke with a child as the main bystander.

**Conclusion:**

The Intervention Mapping approach guided the development of a school-based program to improve Emergency medical services activation in stroke by 12–15 year old children. Our process and approach can serve as a model for researchers and health promotion professionals aiming to improve help-seeking behavior for stroke to improve stroke help-seeking behavior as well as other acute diseases.

## Background

Strokes are one of the leading causes of death with over six and a half million deaths per year worldwide [1] and over 13 thousand deaths in Czechia [2]. In addition, it is the third leading cause of disability worldwide [1], as for every minute of an untreated stroke, up to 1.9 million neurons can be lost [3]. Consequently, even a slight increase in the time from the onset of symptoms to treatment increases the likelihood of disability at discharge [4]. The main reason for the increased time from onset to treatment is the delayed activation of emergency medical services (EMS) by stroke victims or bystanders [5–7], which is mainly caused by the non-recognition of stroke symptoms and delayed or even absent ambulance calls [8–11]. Therefore, there is a need to inform the lay public about the actions that need to be taken to appropriately activate the EMS for stroke.

In reaction to this need, educational campaigns were developed to inform the lay public about EMS activation for stroke in several European countries such as England [9, 12], Sweden [13], Ireland [14] and Germany [15, 16]. Most of them were found to have a positive impact on knowledge about stroke (i.e., symptom and risk factors recognition, intention to call an ambulance) [17]. However, qualitative studies have shown that people who saw such campaigns and knew the main stroke symptoms still failed to correctly identify stroke when it happened in their surroundings [18–20]. The reason for this lies in the complexity of the EMS activation behavior, which in addition to knowledge, is also caused by other essential determinants, such as perceived risk perception, expectations about symptoms severity, social support of significant others, etc [6, 18, 21]. In addition, the results of public education campaigns are highly dependent on intensity, funding and often have a short-term effect [22–25].

These findings highlight the need to develop interventions that address the complexity of the behavior using a systematic and planned approach that will allow program maintenance without excessive retention costs. One possible solution could be shifting stroke education to the school setting. Integrating such programs into the curriculum would allow for continuous implementation without relying on external experts or additional funding. Systematic reviews of stroke school-based programs show that they have the potential to increase the number of individuals capable to recognize stroke and transfer knowledge to other community members [26–28]. However, most of the existing school-based programs suffer from the same disadvantage as programs for the general public - they are focused mainly on stroke knowledge without considering other possible determinants of EMS activation [26]. Therefore, there is a need for evidence- and theory-based school intervention that aims to improve stroke help-seeking behavior.

In this paper, we describe the development of a new version of educational intervention for the Czech e-learning called “HOBIT”, launched at 2014, and aimed to improve emergency medical services (EMS) activation for stroke by schoolchildren aged 12–15 years [29]. E-learning was placed on the HOBIT website and had an educational and an assessment part. Education part consisted of a film. Assessment part consisted of a pretest for measure baseline knowledges and an immediate posttest to give students immediate feedback. The film was done in the health education format by providing information about stroke symptoms, risk factors, appropriate reactions (including calling EMS), and pathophysiology [29]. The targeting of the HOBIT to the specific age group of 12–15 years old children was based on the Czech curriculum, where human biology as well as diseases, injuries and prevention is discussed in several waves in grades 6–9 (lower secondary education) [30].

HOBIT was one of the few school-based stroke awareness programs in Europe at that time [26, 31, 32]. Over 6 years, it was implemented in 182 schools across the Czech Republic. However, recognizing the limitations of knowledge-based approaches and the need to prepare students for real-life situations, we decided to develop a new educational content for the HOBIT e-learning based on behavior change principles. The current study details the planned and systematic development of new educational content for HOBIT using the Intervention Mapping approach.

### Methods

**Fig. 1:**
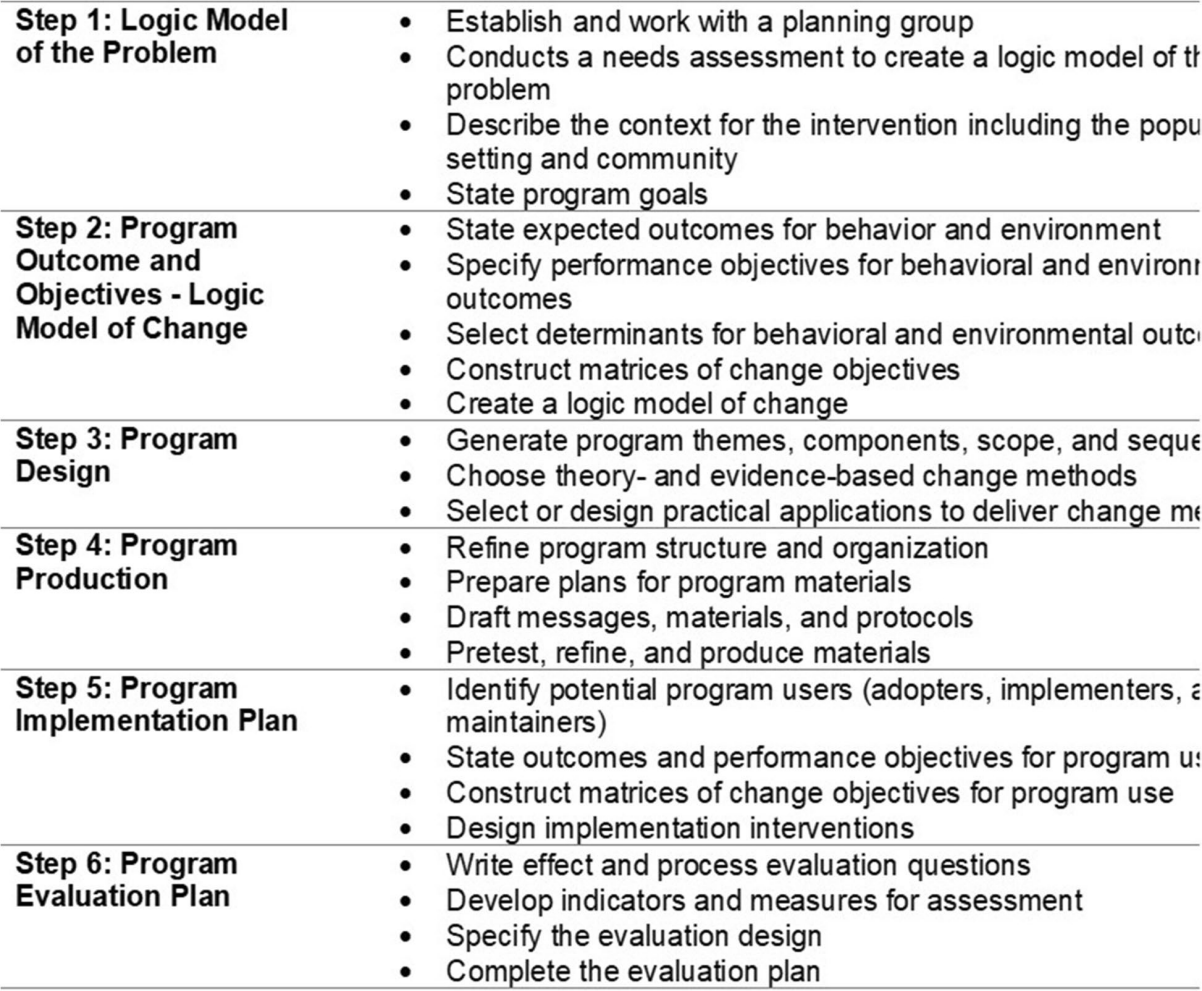
Intervention mapping steps and tasks derived from Bartholomew, 2016 [34]

The Intervention Mapping (IM) approach was used to guide the planned and systematic development of the updated version of the HOBIT intervention. The IM approach provides a framework for effective decision-making at each step of intervention development. The IM approach emphasizes the systematic use of theory and evidence that allows one to address relevant factors causing a health problem and to find the right approach to solve this. An important distinguishing feature is the use of an ecological approach, addressing both individuals, as well as the environment and the participation of all important stakeholders at all stages of the intervention development. IM consists of 6 steps and completion of all of the steps serves as a blueprint for designing, implementing and evaluating an intervention based on theoretical, empirical and practical information [33]. The six steps of the IM process are shown in Fig. 1. In the following sections, each step is briefly described and applied to the development of the HOBIT program.

The first step in the IM approach was to conduct a needs assessment and create the Logic model of the problem. The key purpose of this step was to analyze the health problem and its causes using theory, empirical evidence and available epidemiological data [34]. The problem analysis was performed through three strategies: (1) a literature review of empirical studies, (2) interviews with stroke survivors and (3) an interview with a practicing neurologist.

In the second step, problem analysis was used to determine the behavioral outcomes to be achieved as a result of the health promotion program [34]. In this study, only outcomes that can be reached on the individual level were included. Behavioral outcomes were then divided into several performance objectives, which represent specific steps (i.e., sub-behaviors) that need to be implemented by the target group to conduct target behavior (timely EMS activation). For each performance objective, *personal determinants* were selected. Personal determinants were defined as factors that are under the direct control and influence of the individual and can be changed with interventions [34]. Determinants were selected from the preliminary list of determinants created in the needs assessment phase and based on their changeability and relevance to the intervention development. They were then linked to the relevant performance objectives to create a matrix of change with change objectives, which described what needs to change for the person in order to execute performance objectives in relation to the determinants [34].

In step 3, health promotion specialist conceptualized and designed the intervention. Change methods and applications were chosen at this step. A change method was defined as “a general technique for influencing the determinants of behaviors” ([34], pp. 347). Applications were define as the “ways in which the theory-based methods are presented and delivered in an intervention” ([34], pp. 348). During translating change method into practical applications, the parameters of the method’s efficacy were taken into account [35].

In step 4, the intervention was produced. The program structure and organization, materials, messages, as well as their pretesting and production were also discussed. It was determined where and how potential participants will interact with the program [34]. The appropriate amount of materials and the mode of delivery for a particular school system was also determined [34].

In IM step 5, health promotion specialist “developed an implementation plan to enable adoption, implementation and maintenance of the health promotion program” ([34], pp. 483). However, before embarking on planning for a larger-scale implementation, we aim to evaluate the effectiveness of the intervention in enhancing EMS activation determinants and behavioral intentions. Therefore, in the current project, our focus has been on developing an implementation plan at a smaller scale, specifically for the Randomized Controlled Trial (RCT). After receiving positive results from the RCT, plans for the larger scale implementation will be made. The last step of IM included planning and conducting an evaluation of the intervention [34] which is not a subject of the current study.

## Results

### Step 1: The logic model of the problem

We first formed a planning group comprised of important stakeholders, including the main investigator, health promotion specialist, PR and communication specialist, members of the target group (children), stroke survivors and a neurologist. This planning group was established and coordinated by the Public Health Group of the Stroke Department at the International Clinical Research Center in Brno, Czechia.

The first step was to describe health and quality of life problems related to untreated stroke. The age-standardized stroke years of life lost (YLL) is 864 per 100,000 men and 607 per 100,000 women in Czechia [36]. Disability-adjusted life years (DALY) is 1031 per 100,000 men and 608 per 100,000 women [36]. These YLL and DALY rates are almost two times higher than in Western European countries [36]. Taking into account that the Czech Republic ranks third in Europe in the number of thrombolysis per population [37], the most likely reason for high YLL and DALY rates is delayed ambulance calls by stroke bystanders, which makes it impossible to administer time-dependent treatment to the patient (i.e., thrombolytics).

As the next step, the problem behaviors involved in the inappropriate EMS activation behavior and its determinants were analyzed. To carry out the literature review, the following search terms were used: stroke, awareness, public campaign and pre-hospital delay. Findings indicated that the main reasons for delayed EMS activation are failure to recognize stroke, inability to use the FAST assessment method (Face, Arm, Speech, Time) and call an ambulance [8–11]. The main determinants identified via this approach for the above risk behaviors were: lack of skills in stroke assessment and crisis communication, low self-efficacy in crisis situations, low stroke susceptibility, underestimation of the severity of the situation, inadequate knowledge of symptoms and social influence of significant others who induce a delay or completely forego EMS activation [6, 18, 21, 38, 39].

**Fig. 2:**
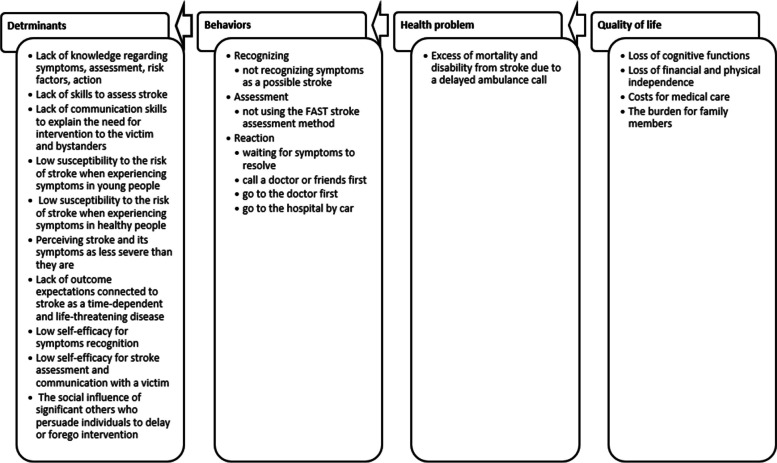
The logic model of the problem (= inappropriate EMS activation for stroke)

Additionally, we conducted one-on-one interviews with two women aged 24 and 29 years old, who had a stroke in their twenties. They were recruited from the stroke ambassadors cooperating with the research team. Their cases were chosen as less typical and less severe cases, which are generally more useful for a deeper understanding of the problem. An interview with a neurologist, who is part of the Stroke Department at the International Clinical Research Center in Brno, Czechia where HOBIT is running, was also conducted to assess the medical perspective on the problem. All interviews were audio-recorded, transcribed and analyzed thematically to identify key themes. The interviews confirmed the results of the literature review by more precisely specifying and describing the specific barriers to EMS activation. Based on the above findings, we created a logic model of the problem, which identified that the main health problem to is the excess of mortality and disability from stroke due to a delayed EMS activation. The main behavior than lead to this problem is: (1) not recognizing stroke symptoms, (2) not using FAST assessment method, (3) incorrect usage of medical services. These behaviors are predicted by several sub-behaviors (determinants), such as lack of knowledge or skills, low stroke susceptibility, low suspected severity, wrong outcome expectations of stroke, low self-efficacy and social influence of significant others. The complete logic model of the problem is summarized in Fig. 2.

### Step 2: Formulating intervention objectives

The main *behavioral outcome* of the HOBIT program is the timely activation of EMS by secondary school children aged 12–15 years old when a stroke is suspected in their environment. This outcome is considered the target behavior in all existing programs for help-seeking behavior in stroke [5, 40, 41].

The selection of performance objectives was based on the F.A.S.T guidelines issued by the American Stroke Association, which distinguishes 3 steps: (1) recognize symptoms, (2) assess them using the FAST method and (3) call an ambulance immediately [42]. These steps have been expanded based on an extensive literature review and interviews with stroke survivors. Therefore, the performance objectives were formulated as follows: secondary school children aged 12–15 years old (1) notice symptoms and compare these with symptoms of stroke, (2) communicate with victim/bystanders and apply stroke recognition methods and (3) call an ambulance and communicate with the operator.

The Logic model of the problem from IM step 1 revealed that important determinants included not only knowledge of stroke, but also risk perception, outcome expectancies, social influence, self-efficacy and skills (see Fig. 2). Their changeability and relevance were assessed via interviews with the planning group along with existing empirical evidence [6, 18, 21, 38]. Those determinants were linked to the relevant performance objectives to create a matrix of change with change objectives. (see Table 1).

**Table 1:** Matrix of change objectives according to intervention mapping recommendations

	Determinants					
*Performance Objectives (PO)*	**Knowledge**	**Risk-perception**	**Outcome expectations**	**Social influence**	**Self-efficacy**	**Skills**
PO1. Notice symptoms and compare these with symptoms of stroke	**K1.1.** Recall stroke symptoms **K1.2.** Recall mimicking symptoms **K1.3.** Recall risk factors	**R1.1.** Recognize younger people are at risk of stroke **R1.2.** Recognize healthy people are at risk of stroke	**O1.1.** Explain that symptoms recognition can save life **O1.2.** Explain that even weak symptoms have severe consequences **O1.3.** Recognize stroke as a severe disease with severe consequences		**SE1.1.** Express confidence in distinguishing stroke symptoms from mimicking symptoms	
PO2. Communicate with victim/bystanders and apply stroke recognition methods	**K2.1.** Recall the FAST method		**O2.1.** Explain that acting assertively can save life	**SI2.1.** Recognize that can intervene even if significant others do not allow it/ it is not allowed by social status	**SE2.1.** Express confidence in applying the FAST method **SE2.2.** Express confidence in communicating that an ambulance call is needed	**SK2.1.** Demonstrate the skills to apply the FAST method **SK2.2.** Demonstrate the skills to explain to victim/bystanders that an ambulance call is needed
PO3. Call an ambulance and communicate with the operator	**K3.1.** Recall calling EMS as the only right reaction **K3.2.** recall what to say to the operator		**O3.1.** Explain that an immediate EMS call can save life	**SI3.1.** Recognize that calling EMS is the correct action even in the presence of social pressure from others	**SE3.1.** Express confidence in the need to call EMS **SE3.2.** Express confidence in describing symptoms	**SK3.1.** Demonstrate the skills to explain the situation to the EMS operator correctly

### Step 3: program design

#### Theory- and evidence-based change methods and practical applications

##### Intervention modality

The initial decision was made regarding the intervention modality. It was decided to keep the e-learning modality of the intervention, as the goal was to update the content of the HOBIT program, which has its own history and a solid network of users throughout the Czech Republic. Here, we define e-learning as education which enables students to acquire new knowledge and skills with the help of educational content available online and provides automatic feedback to the student’s study activities [43].

Just as in the previous version, E-learning is placed on the HOBIT website and has an educational and an assessment part. Such e-learning format was found to be the optimal form for delivering school-based stroke interventions as teachers are not required to have any knowledge about stroke, reduces the burden on teachers to prepare lectures, allows lectures to be given anytime, anywhere. In addition, this format is more suitable for the standard school curriculum, based on the presentation of knowledge and subsequent testing of their assimilation. Therefore, the HOBIT intervention was scheduled as a 20 to 40 min e-learning lesson with a pretest-film-posttest structure, which brings the intervention closer to the usual style of teaching in school lessons.

The reason for choosing video format for the educational part of e-learning was based on the Cognitive Theory of Multimedia Learning which highlights the simultaneous use of visual and verbal methods, thus maximizing working memory capacity and therefore learning outcomes [44–46]. The effective use of video as an educational tool is enhanced with the involvement of three basic elements: (1) cognitive load; (2) student engagement; (3) active learning [46]. Taking into account the above parameters, it was decided to use the Entertainment – Educational (E-E) approach in the video.

E-E incorporates an educational message into the entertainment content [47]. It is considered to be more appropriate than traditional formats for populations with low literacy or motivation, such as children [47]. The is a growing body of scientific evidence suggesting that it is highly effective for changing health behaviors. However, the usage parameters such as narrative involvement (e.g., the level of interest, realism, transplantation) and character identification (e.g., the level of emotional and cognitive identification, similarity, liking) must be met [47].

To assess E-E usage parameters for the specific population of Czech school-children, 17 structured discussions with a total of 157 children aged 12–15 were conducted in November and December 2021. The aim was to assess the preferences of the target group and ensure the socio-cultural relevance of the intervention. During discussions, the form (e.g., cartoon, song, documentary, etc.) and length of the video, as well as other important characteristics (main character, environment), were discussed. Children indicated that they preferred a feature film format with entertainment elements (e.g., jokes or background music) with a length of 2–5 min.

##### Change methods and practical applications

Based on the modality of the intervention (E-E video), determinants, and change objectives, behavior change methods were chosen. In our case, we selected the change methods based on those formulated by Kok et al. [35]. The health promotion specialist identified specific practical applications for the intervention by matching change objectives with theoretical methods and their parameters for application. For example, for the change objective “recall stroke symptoms,” the “elaboration” change method was chosen. The parameters to elaboration effectively change target behavior are personal relevance, surprising, repeated, easily understandable, and direct instruction. To incorporate the mentioned change objective with its change method to the intervention, the practical application “knowledge about stroke is given in personally relevant, surprising and repeated way by embedding it into entertainment storytelling” was created. Specific practical applications of the determined change objective are shown in Table 2.

Practical applications were then incorporated into a design document as recommendations for script processing by a production company. The specific artistic and visual processing of the practical applications was left to the discretion of the production company (see step 4 “Program Production”).

**Table 2:** Pairing the relevant determinant with Behavior Change methods to target in intervention

Determinants and their change objectives	Method	Parameter	Practical application
Knowledge - recall stroke symptoms - recall mimicking symptoms - recall risk factors - recall the FAST method - recall calling EMS as the only right reaction - recall what to say to the operator	-Information -Elaboration -Providing cues	Attention, reinforcement, identification, self-efficacy, coping, personal relevance, surprising, repeated, easily understandable, direct instruction, tailored selection	-Knowledge about stroke is incorporated into a film script and repeated at the end of the film -Knowledge about stroke is given in personally relevant, surprising and repeated way by embedding it into entertainment storytelling with a child as a main character -Real-life situations are used as cues in the film script
Risk-perception -recognize younger people are at risk of stroke -recognize healthy people are at risk of stroke	-Scenario-based risk information -Belief selection	Attention, reinforcement, identification, self-efficacy, coping Scenario with a cause and an outcome Investigation of the current beliefs	-The model in the film presents a scenario that demonstrated that stroke can also happen in the absence of diseases that may cause stroke - The belief that stroke only happens to old people with pre-existing conditions such as obesity, hypertension, or diabetes, needed to be changed. The belief that stroke could happen to all categories of people is introduced in the film
Outcome expectations - expect that symptoms recognition can save life - expect that even weak symptoms have severe consequences - expect that acting assertively can save life - expect that an immediate EMS call can save life	-Modeling -Elaboration -Cultural similarity	Attention, reinforcement, identification, self-efficacy, coping, personal relevance, surprising, repeated, easily understandable, direct instruction, use socio-cultural characteristics of the target group	-The main character decides to help a woman with a stroke because he knows about the consequences of stroke and the role of time in stroke treatment - The message about stroke outcomes is given in a personally relevant surprising way by embedding it into entertainment storytelling with a child as a main character -The film is tailored for the target group (children) by giving a message in a social-cultural relevant context
Social influence -recognize that the witness can intervene even if significant others do not allow it / it is not allowed by social status - recognize that calling EMS is the right action even in the presence of social pressure from others	-Modeling - Belief selection	Attention, reinforcement, identification, self-efficacy, coping	-The main character (a child) resists the social influence exerted by significant others (i.e., the father and the police) and takes appropriate action despite their disagreement -The belief that children lack the ability to act independently in urgent situations and that their knowledge is not valid, is changed in the film. The capacity and empowerment of children to act in urgent situations are promoted by peer modeling
Self-efficacy - express confidence in distinguishing stroke symptoms from mimicking symptoms - express confidence in applying the FAST method, - express confidence in communication that an ambulance call is required - express confidence in the need to call EMS - express confidence in describing symptoms	-Modeling	Attention, reinforcement, identification, self-efficacy, coping	-The primary model is portrayed as a peer who successfully overcomes the barriers to seeking emergency medical care for stroke. -To enhance identification with the character, the film highlights the common peer characteristics of the protagonist.
Skills -demonstrate the skills to apply the FAST method -demonstrate the skills to explain to victim/bystanders that an ambulance call is needed - demonstrate the skills to explain the situation to the EMS operator correctly	-Modeling	Attention, reinforcement, identification, self-efficacy, coping	-The film demonstrates the application of the FAST method in a high-stress situation, providing children with real-life examples of how the method must be used. - The film demonstrates communication between the principal character (child) and other characters who are actively discouraging him from EMS activation. In doing so, the film effectively highlights the essential skills of crisis communication in the context of stroke help-seeking behavior.

### Step 4: program production

#### Prepare design documents, draft message and materials

The health promotion specialist prepared a design document, appropriate for film creation. In the first part of the document, the length and the format of the product, the description of the audience, the way the users will interact with the product, and the expected impact were described. In the second part of the document, determinants, performance objectives, change objectives, methods for behavior change and their parameters have been detailed (see Table 2).

For the incorporation of the above elements into the film, a production company with experience in health-related topics, children-related content and entertainment-education format was hired. Based on the design document, the production company wrote the script showing the creative translation of scientific elements into the film. The health promotion specialist ensured that all elements were incorporated into the film according to the design document. In the first version of the script, the health promotion specialist made corrections to the symptoms, their strength and sequence, the display of risk factors as well as the inclusion of some other determinants.

#### Pretest, refine and produce the program

Before the final production, the draft of the script was pretested with 15 children aged 12–16 (mean age 14, 47% of females) to ensure that all the parameters of effectiveness were met. Children were recruited via email to the school principals cooperating with the project team requesting voluntary completion of the questionnaire by interested students subject to informed consent from parents or legal guardians. Pretest had the format of a questionnaire with open-ended and close-ended questions. Considering the parameters of the change methods used, the following dimensions were measured: interest, realism, similarity with a character, cognitive and general involvement and emotional and cognitive identification. They were measured on the 5-point Likert scale (1-strongly agree, 5-strongly disagree) using appropriate statements [48–51]. These results were supplemented with informal feedback collected in open-ended questions (e.g., “What could we change in the scripts to make it more appealing to you?”).

The percentage of participants who agreed or strongly agreed with the statements was 73% (*N* = 11) for interest, cognitive involvement and cognitive identification; 67% (*N* = 10) for general involvement; 67% for realism (*N* = 10); 40% (*N* = 6) for similarity and emotional identification with the character. Informal feedback revealed that the main reason for lower scoring on the latter dimensions was the use of a master model rather than a coping model, usage of which is one of the main parameters for modeling as a behavior change method. Other identified reasons included unrealistic emotional expressions and the communication style of the characters.

**Fig. 3:**
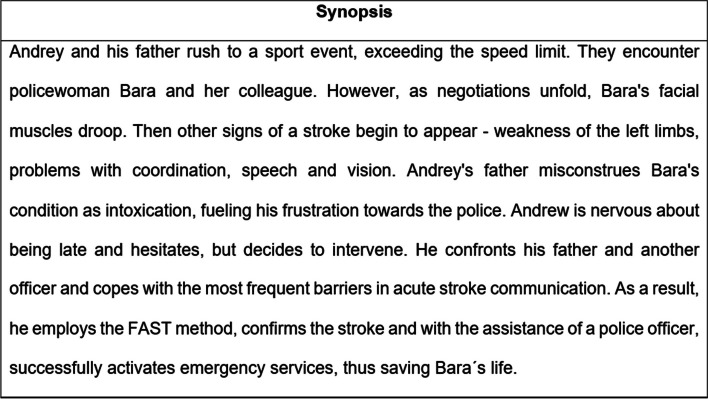
Synopsis of the E-E film for the HOBIT project

Based on the above results, health promotion specialist proposed several changes in the script to improve cultural relevance in terms of emotional expressions, communication styles and identification with the characters [52]. Proposed changes included: (1) incorporating coping model by creating scenes in which the main character experienced doubts about intervening in the situation and received assistance from adult characters, (2) change the father and police character’s behavior to be more polite and empathetic to make it look more realistic. The revisions were incorporated into the final version of the script by the production company. The synopsis of the film script is shown in Fig. 3.

**Fig. 4:**
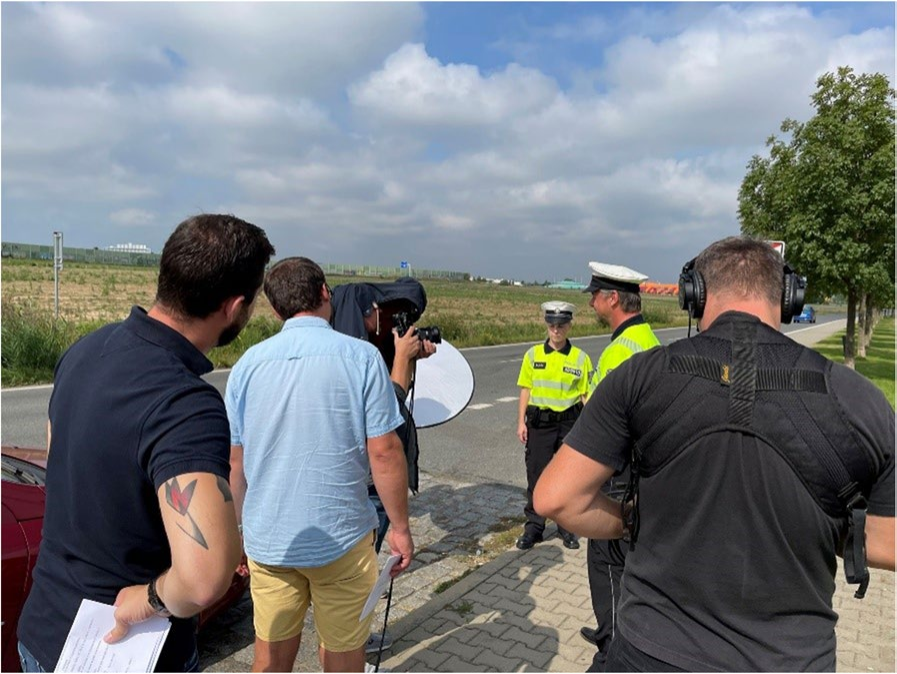
The filmmaking process of the E-E film for the HOBIT project

**Fig. 5:**
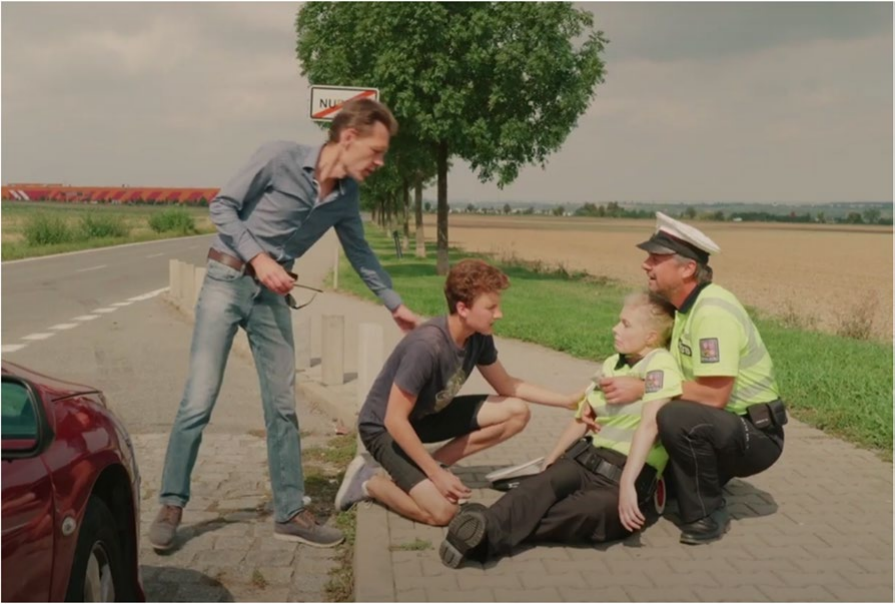
The screenshot of the E-E film for the HOBIT project

The filmmaking process involved members of the production company as well as members of the working group. The film was shot in Prague over two days, and professional actors were hired by the production company and approved by a PR specialist and a health promotion specialist. When making the film, the director followed the script (a short version is shown in Fig. 3 Synopsis). During filming, a PR specialist and a health promotion specialist checked that all the practical applications specified in step 3 were followed. The filmmaking process is shown in Fig. 4. Subsequently, the production company edited the film so that its final length did not exceed 5 min. A health promotion specialist subsequently approved the final version of the film. As a result, a 5-minute narrative film in the entertainment-education format modeling an acute stroke with a child as the main savior was created. The screenshot of the film is shown in Fig. 5. The film’s final version was integrated as an intervention component into the e-learning platform on a dedicated website for the HOBIT program.

### Step 5: Implementation plan

#### Implementation plan for RCT

School management may be considered as an important stakeholder in school-based interventions. However, past experiences with the implementation of the initial HOBIT intervention show that contacting school principals has proven to be ineffective, as they either do not respond to the invitation or refer to teachers. Moreover, the curriculum in the Czech Republic additional subject areas in which the content is determined by the teachers themselves. Therefore, in the current intervention, school teachers serve as the adopters and implementers since they are potentially interested in incorporating external programs into their lessons.

The primary outcome of the adaptation that a minimum of 2 schools located in the South-Moravian region, Czechia, would adopt the HOBIT program in 2023. In this case, the performance objectives for adaptation include that teachers should: (1) evaluate the need to implement the program, (2) review all the information regarding the program and (3) decide to implement the program in their lessons.

As for the implementation, the main outcome is that the HOBIT program is implemented including all of its components (i.e., pretest-film-posttest) in the predetermined e-learning form according to the instructions. In order to consider the implementation to be successful, there are several performance objectives to be met: (1) prepare the computer classroom, (2) make sure all students have successfully registered at the e-learning website and (3) make sure that all students complete all components of e-learning. In regard to facilitating the implementation with high completeness and accuracy, special video instructions have been developed and posted on the website. Teachers will also be provided with free lessons concerning stroke and provided with contact for the technical support line.

If RCT demonstrates a positive effect of the HOBIT intervention on changing EMS activation determinants, a large-scale implementation plan including matrices of change objectives, change methods and practical applications will be developed. In such a case, an implementation plan will also be developed for program maintenance, ensuring the program continues over time and becomes institutionalized (i.e., integrated as part of the organization’s routines) [34].

### Step 6: Evaluation plan

We used a clustered randomized controlled trial (RCT) to assess the efficacy of the HOBIT intervention on EMS activation in suspected stroke. As was mentioned in step 2, the main behaviors related to appropriate EMS activation for stroke are noticing symptoms and comparing these with symptoms of stroke, communicating with victim/bystanders and applying stroke recognition methods, calling an ambulance and communicating with the operator. However, measuring help-seeking behavior in acute diseases at the behavior outcome level is challenging [41]. Therefore, the effect evaluation of the current intervention was measured on the behavior intention and determinants’ level as the difference in mean test scores before and after the educational film. The results of the RCT plan will be published elsewhere.

## Discussion

This paper presented the detailed stepwise process used to develop the HOBIT school-based intervention aimed to improve EMS activation for suspected stroke by 12–15 years old children. This study contributes to the literature as the first one to design a stroke intervention for this specific target group using the systematic intervention development framework – the Intervention Mapping approach. The development process resulted in an entertainment-educational video modeling an acute stroke and adjusted for the target group’s psychosocial and cultural specifics.

The added value of this study lies not only in the creation of a theory- and evidence-based intervention, but also in the systematic documentation of its precise content, illuminating the development process. The development of interventions remains a ‘black box’ within the complex intervention trial design and is rarely published. Thus, researchers are often left without guidelines and tend to repeat methodological mistakes found in previous studies, which leads to some interventions never having an impact on public health [53].

Through a deep multi-method needs analysis, we compiled a more accurate list of determinants that affect EMS activation that include including not only knowledge [32, 54–57], but also social influence, risk perception, self-efficacy, outcome expectations and skills. These determinants were combined with sub-behaviors to create the most precise change objectives, for which tailored behavior change methods were then applied. To our knowledge, most existing interventions do not use specific behavior change methods or do not clarify their use [32, 54–56, 58].

As a result, we can assert with greater confidence that the HOBIT intervention is: (i) based on extensive formative work, stakeholder engagement and “best practice” evidence from evaluated interventions; (ii) combines theory with scientific evidence to inform the intervention objectives; and (iii) embedded within Czech school curriculum to facilitate broader scaling. Therefore, the present study makes a valuable contribution to the limited literature on the development of effective interventions to improve EMS activation for suspected stroke by school children.

While this study highlights the promise of the HOBIT intervention, further research is warranted to explore its effectiveness in larger-scale implementation. Therefore, we plan to implement the program at the level of the entire Czech Republic as the next step. Additionally, since one of the stated benefits of targeting stroke programs at children is that they spread their knowledge to the wider community, we plan to evaluate spreading the impact of HOBIT on the family members of participating children. Furthermore, exploring the cost-effectiveness of implementing the HOBIT intervention within the school curriculum could inform future public health decisions.

## Conclusion

This paper reports the development of the school-based HOBIT program aimed to improve EMS activation for suspected stroke by 2–15 years old children. It represents the first attempt to systematically apply theory and evidence in the development of an intervention of this nature and was designed to reduce mortality and disability rates caused by delayed EMS activation among stroke bystanders. Intervention mapping was a useful planning approach to the development of the current project and helped to create a more tailored intervention with increased potential to change EMS activation behavior. The data shown in this article can provide valuable experience for designing and refining further interventions.

## Data Availability

All data generated or analyzed during this study are included in this published article.

## Abbreviations

EMS: Emergency Medical Services
IM: Intervention Mapping
FAST: Face, Arm, Speech, Time
E-E: Entertainment-Education
YLL: years of life lost
DALY: Disability-adjusted life years
RCT: Randomized Controlled Trial
